# Predictive Mechanistic Model of Creep Response of Single-Layered Pressure-Sensitive Adhesive (PSA) Joints

**DOI:** 10.3390/ma14143815

**Published:** 2021-07-08

**Authors:** Hao Huang, Abhijit Dasgupta, Narendra Singh

**Affiliations:** 1 Department of Mechannical Engineering, University of Maryland, College Park, MD 20740, USA; dasgupta@umd.edu; 2 Microsoft Corporation, Redmond, WA 98052, USA; narendra.singh@microsoft.com

**Keywords:** pressure-sensitive adhesive, creep, mechanistic modeling

## Abstract

This paper explores the uniaxial tensile creep response of acrylic-based pressure-sensitive adhesive (PSA), which exhibits a unique multi-phase creep response that does not have the classical steady-state region due to multiple transitions caused by several competing mechanisms: (i) cavity nucleation and growth in the interior of the adhesive material of the PSA system, as well as at the interfaces between the PSA and the substrate; (ii) fibrillation of the bulk adhesive, and (iii) interfacial mechanical locking between the adhesive and the bonding substrate. This results in multiple regimes of strain hardening and strain softening, evidenced by multiple regions of steady-state creep, separated by strong transitions in the creep rates. This complex, multi-phase, nonlinear creep response cannot be described by conventional creep constitutive models commonly used for polymers in commercial finite element codes. Accordingly, based on the empirical uniaxial tensile creep response and the mechanisms behind it, a new mechanistic model was proposed. This is capable of quantitatively capturing the characteristic features of the multiphase creep response of single-layered PSA joints as a function of viscoelastic bulk properties and free energy of the PSA material, substrate surface roughness, and interfacial surface energy between the adhesive and substrate. This is the first paper to present the modeling approach for capturing unique multi-phase creep behavior of PSA joint under tensile loading.

## 1. Introduction

**Figure 1:**
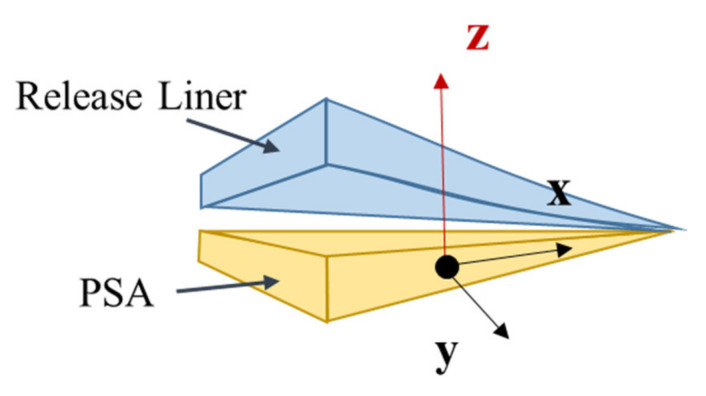
Schematic of single-layered PSA and loading direction.

This study examines the creep response of PSA-bonded joints to tensile (peel) loading, because such joints creep more severely under tensile loads (in thee *zz* direction in Figure 1) than under shear loads (in the *xy* plane, in Figure 1). The PSAs discussed in this study are commercially available acrylic-based PSAs. The specific identities of the PSAs are not allowed to be listed, but the generic behaviors of most PSAs are very similar. The literature on creep behavior of PSA materials and PSA-bonded joints is discussed below, first for shear loading and then for tensile loading.

Many studies on the tack, peel, shear, and shear creep properties of PSA bonded assemblies have been performed in recent decades. Fujita et al. studied the effects of miscibility and viscosity on the shear creep resistance of PSAs based on natural rubber. They concluded that the holding time (which is the required time for the PSA tape under shear load to completely debond from the adherend) tended to decrease as the tackifier content increased [1]. Sosson et al. investigated the shear failure mechanisms of PSA and found that for a weak cross-linked adhesive, failure occurs by creep rupture, while for a highly cross-linked adhesive, failure is caused by fracture [2]. Kim et al. tested hot melt PSAs with different viscosities and found that the shear creep resistance increased as the PSA viscosity increased [3]. Poh and Kwo investigated the effects of adhesive (coating) thickness on shear performance of Standard Malaysian Rubber (SMR)-based PSA. They concluded that the shear strength increased as the adhesive thickness increased [4]. Zosel studied the correlation between the shear strength and the mechanical properties of PSA by measuring the deformation behavior in a static and dynamic shear test. He noticed that the static shear strength and holding time (time to failure) of the sample can be calculated from the master curve of the dynamic shear test, but the method cannot be applied to a highly viscous polymer [5]. Czech demonstrated how crosslinked could affect PSAs’ mechanical performance [6]. Martin and Derail have studied the relationship between rheological and peeling properties for hot-melt PSAs based on homopolymers or copolymers blended with tackifying resins [7]. Poh and Yong performed a study to understand the dependences of shear strength of ENR-based adhesive on the molecular weight of the rubber. The observation concludes with the appropriate molecular weight range for obtaining the optimal shear strength of this PSA [8]. Lee et al. studied the effect of crosslink density of acrylic PSAs on their mechanical performance. The research indicates that there is non-monotonic dependence of PSA adhesion and flexibility on crosslink density [9]. Additional studies have advanced the understanding of the effects of new candidate formulations and processes for PSAs performance. Czech et al. reported the effect of novel suitable for addition photoinitiators on peel, tack, and shear strength of acrylic PSAs after UV cross-linking [10]. Sancho-Querol et al. explored a new formula with blended ECH resin that enable PSAs exhibiting excellent creep, high tack, high peel strength, and lap-shear strength at room temperature [11].

Creep of PSA-bonded joints under tensile loading appears to depend not only on the PSA material itself but also on the substrate that the PSA is bonded to. Hait and Barthel proposed a model for evaluating the effect of substrate surface roughness on adhesive bonding under tensile loading [12]. Their model provides approximate analytical expressions for the sensitivity to surface asperity and exhibits the full viscoelastic adhesive contact phenomenology such as stress relaxation inside the contact zone and creep at the contact edge. Yamaguchi et al. proposed a simple “block” model that can capture the major characteristic features of stress-strain curve from a tack test (tensile loading) [13]. However, their model does not have enough control over the cavitation or interfacial cavity growth and can only be applied for the stress–strain response, not for creep response. Hao et al. investigated the creep performance of PSA joints under out-of-plane loading and reported a number of preliminary results [14,15].

In summary, the published data on creep of PSA joints focus mostly on the understanding of shear creep performance. In contrast, the creep resistance to loading in the out-of-plane loading direction (peel or *zz* direction, as shown in Figure 1) has not received enough attention. Compared to in-plane loading, *xx*, *yy*, and *xy*, loading along the peel direction (*zz*) shows the most damage for this kind of adhesive joint structure. In practice, this kind of loading can be caused by external multiaxial loading and initial curvature (warpage) mismatch of bonding substrates. Even the residual stress from a small geometric mismatch can pose a hazard for the creep performance of the PSA layer in a long-term application. 

The primary goal of this study is to report test results and a mechanistic model for the tensile creep response of single-layer PSA joints. The motivation of this study is to understand the deformation of a PSA joint in the field due to long-term static loading introduced by the curved substrates that it is bonded to. Any out-of-plane misalignment or strain relaxation of the curved substrate can result in a tensile stress loading on the adhesive layer. Thus, the uniaxial creep performance of a PSA joint consisting of two rigid substrates bonded with a single-layered PSA material has been empirically investigated under different loading conditions. The joint variables include the PSA system, the substrate material, and substrate surface roughness. Based on the empirical observations and measurements, we propose a mechanistic model that can predict the unique multi-phase tensile creep response as a function of adhesive properties (such as modulus and viscosity), as well as substrate properties (such as surface free energy and surface roughness). The utilization of such modeling is not only for design and virtual testing but also, for real-time prognostic health management (PHM). PHM application requires continuously updated predictions of the residual creep life of adhesive joints and is enabled by using such models as “digital twins” by continuously re-calibrating them with real-time in situ data (such as strain, stress, and temperature).

## 2. Experiments

**Figure 2:**
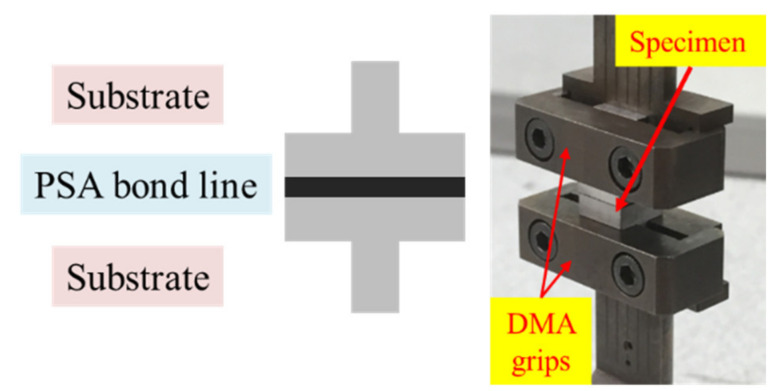
Uniaxial test sample and fixture of the tester.

The test specimen consists of two rigid T-shaped aluminum substrates bonded with a single-layered PSA, as shown in Figure 2. Both substrates were mounted between tensile loading grips of a Dynamic Mechanical Analysis (DMA) tester for constant stress uniaxial creep tests in the tensile or peel (*zz*) direction. The size of the PSA bond line was 7 mm×7 mm with a thickness of 0.05 mm and 0.13 mm. To understand the effect of the loading condition, different stress levels, 50 and 70 kPa, were used. The accelerated test method was used to evaluate the PSAs creep performance. An elevated temperature of 70 °C, which is the maximum temperature that the PSA joint can be exposed to in field applications, was selected for the creep testing. Substrates with different surface roughness were prepared by sanding the surface with various grades of sanding paper, and surface roughness was measured by Atomic Force Microscope. Detailed procedures of sample preparation and measurement are described in a previous stress–strain study [16].

**Figure 3:**
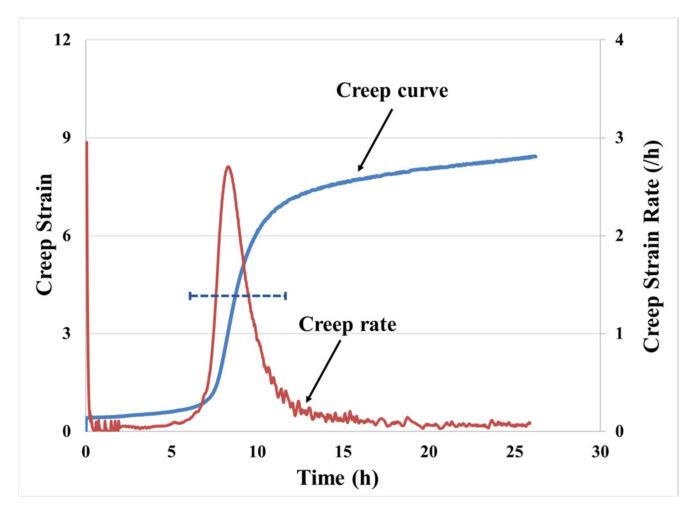
Creep test results of single-layered PSA under 50 kPa and 70 °C.

A creep result, which consists of multiple steady-state (secondary creep) regions separated by sharp peaks and transitions in the creep rates, under test conditions of 50 kPa and 70 °C, is shown in Figure 3. This behavior is unlike that in metals and traditional polymers, which demonstrate a single steady-state region. Eight percent of the observed creep response was dominated by the multi-phase secondary creep regions, which are shown in Figure 3 (primary creep only lasted for a few mins and is hard to observed from the creep curve). The peak rates during the transitions in this multi-phase secondary region may have more than 6000% change compared to the steady-state creep rates. With this applied loading condition, the transitions lasted for about 25% of total creep time (total creep time is defined as the time from the initiation of the creep test to the sample entering tertiary creep, which is about 25 h for this) and accounted for more than 45% of the total creep strain (accounting for almost 600% of the accumulated strain). The dashed line indicates the variability of time to creep transition in the secondary creep region. The mean and standard deviation in terms of strain to critical locations of the creep curve are listed in Table 1. The creep resistance of a PSA joint is found to be more sensitive to the interfacial bonding quality than its pull strength [14,15,16,17], while the strain to critical location of the creep curve is more consistent across measurements. In other words, the variability in the creep rate seen in tests under identical macro-loading conditions is significantly larger than in stress–strain tests. When PSA is bonded to two rigid substrates, a random amount of air pockets can be trapped at the bonding interface. The trapped air at the bonding interface reduces the effective bonding area. This phenomenon can be found from some other transparent substrates such as glass. The reason for the variability in the creep test is suspected also due to the voiding at the interface is. However, this has not been verified in the current study.

These transitions in the creep curve are believed to be a result of the same underlying physics that produced the transitions in the tensile stress–strain tests reported elsewhere [16]. The multiple phases are the result of competition between the mechanisms of cavitation (in the bulk of the adhesive as well as at the interface between the adhesive and substrate) and fibrillation of the bulk adhesive. A significant difference between the stress–strain test and the creep test is the critical stress needed for initiating the transition. Usually, the initiation of cavity growth in stress–strain tests is associated with high stress level [14,15,16], which is well in excess of the creep stress used in this study. The apparent stress level in a creep test is usually significantly lower than the critical stress for cavity nucleation predicted by Gay and Leibler’s model [18]. Cavity initiation under such low stress levels during creep tests is speculated to be assisted by the diffusive motion of polymer molecules and accelerated by stress. The initial cavity growth is slow at the initial stages of creep. As the initial size of defects reaches a threshold value, the force balance around the cavity exceeds the threshold levels and the system becomes unstable, causing the cavity to start growing rapidly, resulting in the rapid increase in creep rates seen during the sharp transitions.

**Figure 4:**
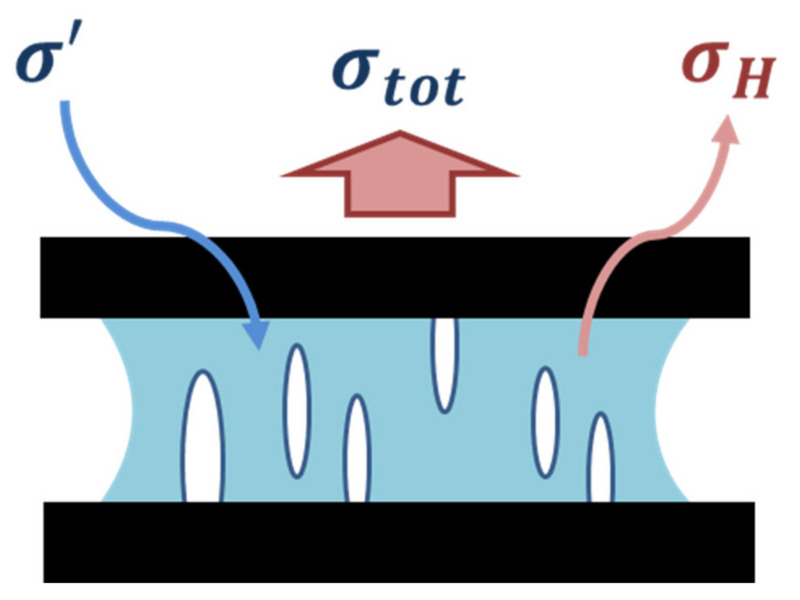
Schematic of change in hydrostatic $\sigma_{H}$ and deviatoric $\sigma^{\prime }$ stress due to the mechanism of cavitation, cavity growth, and fibrillation. Cavity initiation and growth decreases the geometric confinement of the adhesive layer, thus decreasing $\sigma_{H}$ and allowing a compensatory increase in $\sigma^{\prime }$.

Cavitation and growth of bulk and interfacial cavities were extensively discussed in our previous paper [16].These mechanisms associated with such phenomena result in thin fibrils that can decrease the confinement, leading to a release of hydrostatic stress stored in the adhesive layer. Since the creep deformation is a stress-controlled process and the total stress is equal to the summation of deviatoric stress and hydrostatic stress, decreasing the hydrostatic stress leads to a compensatory increase in the deviatoric stress inside the bulk PSA, as shown in Figure 4. The rising deviatoric stress results in a change in the creep rate during the creep debonding process, which becomes a run-away, self-accelerating process as the relative growth rates of the cavities increase and fibril cross-sections start to decrease.

### 2.1. Effect of Joint Geometry

**Figure 5:**
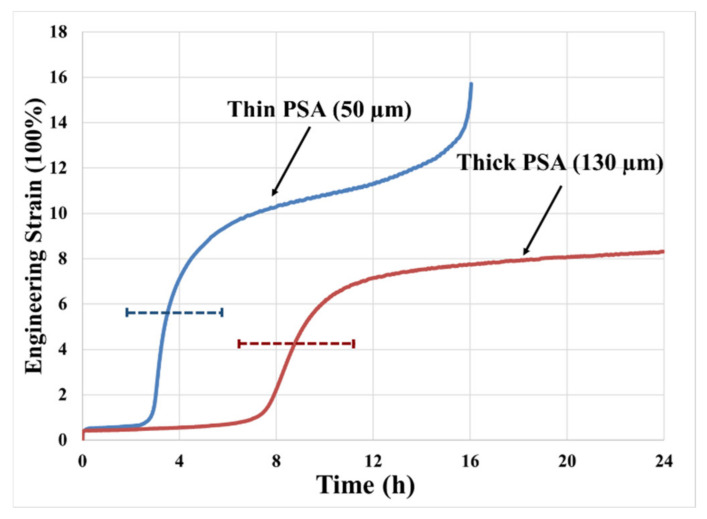
Creep response of thin vs. thick PSA under loading conditions of 50 kPa and 70 °C.

The creep performance is not only influenced by the loading conditions but also affected by the geometry of the joint. For example, the creep resistance of the joint increases as the PSA layer thickness decreases. Figure 5 displays the creep curve of a 50 µm thick PSA vs. a 130 µm thick PSA joint (both joints use the same PSA material). Compared to the thin sample, it takes twice as much time to initiate the cavitation in the thicker sample. Geometric confinement is higher in the thinner PSA than in the thicker PSA. Therefore, the accumulation of hydrostatic stress is much easier in a thin sample than in a thick sample. Under such circumstances, the hydrostatic stress in the thin carrier layer before cavity growth initiation is higher than in the thick carrier layer. This higher hydrostatic stress can result in earlier cavitation in the adhesive layer. In addition to our empirical observations, Tordjeman et al. concluded that the actual bonding area of a PSA joint bonded with thinner PSA is less than a joint bonded with thick PSA (under the same bonding conditions and same substrate and adhesive properties), which is attributed to insufficient adhesive flow into the microscale pits and valleys of the rough surface of the bonding substrate (leading to the larger size of interfacial voids and smaller effective bonding area) [19]. This observation is also verified by our experimental data—both larger interfacial void and higher hydrostatic stress lead to early cavitation of thin PSA joint. The effect of adhesive thickness on actual bonding area saturates as the PSA becomes too thick, and the effect is also dependent on the material properties of PSA and substrate surface properties.

However, the thickness of adhesive does not always help the creep resistance of a PSA joint—it is the result of constant competition between the actual bonding area and geometric confinement of the PSA joint. The aspect ratio of the area to the thickness of the adhesive layer (and hence the geometric confinement in the adhesive layer) decreases as the thickness of the adhesive layer increases. Thus, a thicker PSA joint can creep faster because its stress field is dominated by the deviatoric part, in comparison to a thinner PSA joint under the same loading condition. The empirical part of this study does not include the effect across many adhesive thicknesses due to the limitation of sample availability. However, the predictive model has the capability built in to capture the effect of adhesive thickness on creep by calibrating the parameter of penetration depth (*α*) of adhesive to fit the data from experiments and/or literature. 

### 2.2. Effect of Loading Stress Level

**Figure 6:**
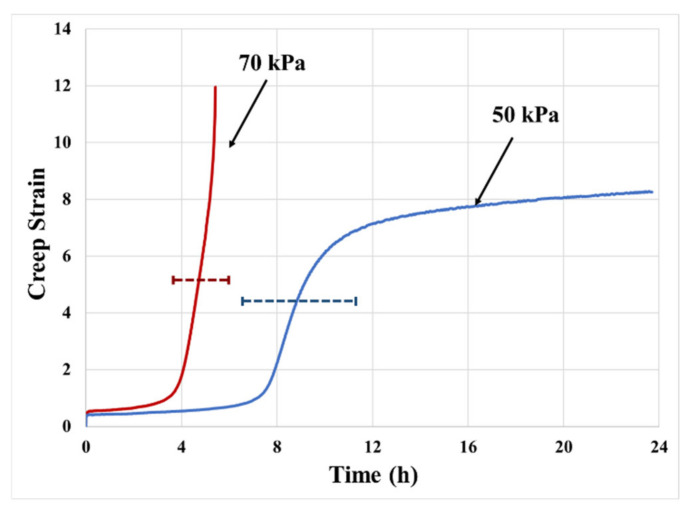
Effect of loading stress level on creep curve at 70 °C (130 μm thick PSA).

Figure 6 shows the creep test results at stress levels of 70 kPa and 50 kPa. Increasing creep stress from 50 kPa to 70 kPa raises the creep rate and shortens the time of transition to the next steady-state region, due to quicker release of hydrostatic stress after cavitation (higher hydrostatic stress leads to higher cavity growth rate). Higher stress around the cavities increases the expansion rate of the cavity and decreases the duration of the transition process. Therefore, the average creep rate in high-stress conditions is twice as much as in the low-stress condition.

### 2.3. Effect of Substrate Surface Properties

**Figure 7:**
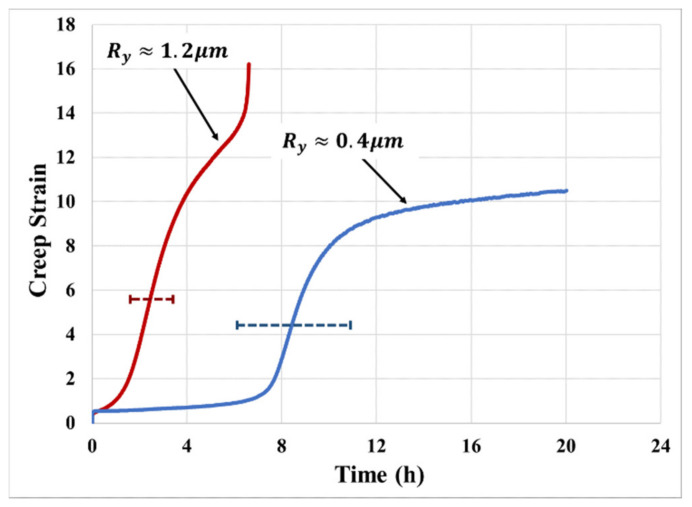
Effect of surface roughness on the creep responses of PSA bonded with aluminum substrate with different surface roughness (130 μm thick PSA).

The choice of substrate affects the interfacial cavitation process and hence alters the creep response, just as it was seen to affect the stress–strain response [16]. The key features of the substrate are interfacial wetting (characterized by the interfacial surface energy) and surface roughness (*R_y_*). As an example, Figure 7 shows the effect of surface roughness on the creep of PSA joints. In this respect, the overall creep resistance decreases by about 70% as the surface roughness increases. The decrement on creep resistance is calculated based on the total time to tertiary creep: with rough substrate, $R_{y}\approx 1.2\;\mu m$, and time to tertiary creep is 6 h; with less rough substrate, $R_{y}\approx 0.4\;\mu m$, and time to tertiary creep is 20 h. The PSA bonded with a higher surface roughness substrate reaches the primary transition in a very short time. This is due to the highly rough surface resulting in larger initial defects, which lowers the critical strain energy needed to initiate the cavitation process. Surface roughness was seen to produce a non-monotonic effect on the stress–strain behavior, as shown in our previous work [16], but the creep results obtained in this study are not comprehensive enough to explore similar non-monotonic trends in the creep response. However, the mechanistic predictive model for creep response, presented in the next section, does indicate that creep response should have a non-monotonic dependence on the surface roughness of the bonding substrate, since the same trade-off between effective bonding area and initial micro-void size also applies in the creep deformation mechanism.

## 3. Predictive Mechanistic Creep Model

Creep deformation is modeled using the same “block” model that was used to model the stress–strain response in PSA joints in our previous work [16]. The deformation is based on similar debonding mechanisms, which are bulk cavitation, interfacial cavitation, cavity growth, fibrillation, and interfacial slippage between adhesive and substrate. Competition between these mechanisms results in different rates of released hydrostatic stress stored in the bulk PSA and change in effective structural stiffness (softening vs. stiffening) of the joint, thereby resulting in changes in effective creep resistance. The creep tests of the PSA discussed above are simulated here, using this virtual testing model, to verify the ability of this model to reproduce the creep behavior observed in the test results.

**Figure 8:**
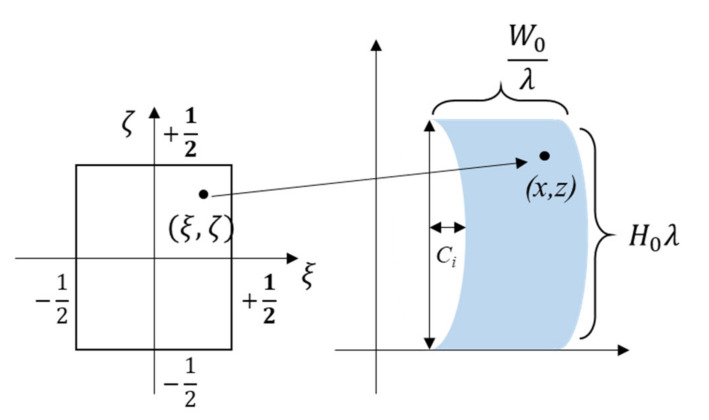
Material coordinate and spatial coordination to describe the block motion and deformation [13].

The creep deformation model is adapted from Yamaguchi’s “block” model [13]. Yamaguchi developed a “block” model for stress–strain behavior of single-layered PSA, and we reported on further enhancements and improvements we brought to that model for stress–strain behavior [14,15,16]. This enhanced “block” model is further improved in this paper, to model the creep response. Details of the “block” model were presented earlier by the authors [16], but relevant features are reviewed here for completeness. The elongation deformation is defined as the stretch ratio λ, and the global transverse deformation caused by Poisson’s effect is described by parameter *C_i_*, as shown in Figure 8. Deformation of each block during the loading process is assumed to be parabolic. The material point (ξ, ζ) moves to the spatial position (*x, z*), as described by the equations below [13].

(1) $$x=X_{i}+\frac{W_{0}\xi }{\lambda }+\frac{C_{i}}{3}\left(1-12\zeta^{2}\right)$$

(2) $$z=H_{0}\lambda \left(\zeta +\frac{1}{2}\right)$$

where $X_{i}$ is the location of the center of mass of block *i*, *C_i_* is the parabolic value of block *i*, and *W*_0_ and *H*_0_ are the initial width and height of the same block *i* and λ is the stretch ratio.

Most of the PSAs of interest in this study are highly ductile and capable of cavitation and fibrillation and can deform by more than 1000% of their original shape. Therefore, Green’s strain is used to describe the stretched PSA as it is accurate over large strains. The specific form of the Green strain tensor and the corresponding velocity gradient for this 2D problem were defined in our previous study [16].

**Figure 9:**
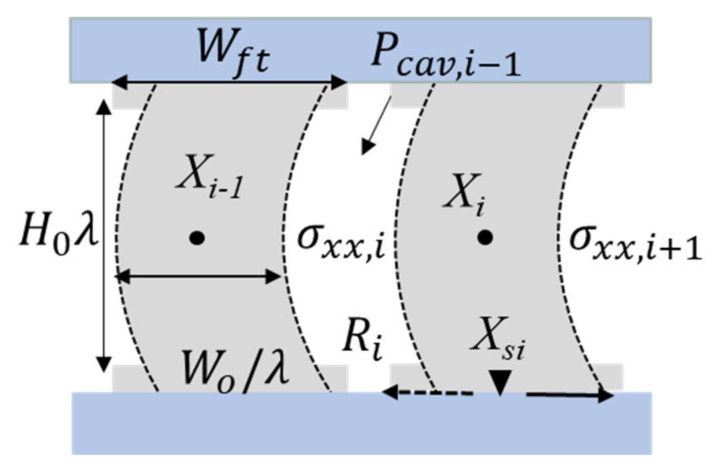
Force balance of *i*-th block.

In the current block model (according to previously reported data [14,15,16]), there is hour-glassing deformation of the fibril, due to mechanical locking at the substrate interface. The width of the fibril body follows the volume conservation of an incompressible material (the bulk PSA is modeled as an incompressible material), while the evolution of the contact length between the fibril foot and substrate follows a detachment model for stretched viscoelastic fibril proposed by Glassmaker [20]. The simplified geometry of the fibril bulk and the fibril foot was discussed in the previous paper on the stress–strain response [16] and has been repeated here in Figure 9 for completeness. At the same time, the corresponding equations for force balance, cavitation in the interior of PSA (Rayleigh–Plesett model), interfacial cavitation, cavity growth, and fibrillation are given in the same paper [16] and omitted here for brevity.

### 3.1. PSA Material Constitutive Model for Creep

**Figure 10:**
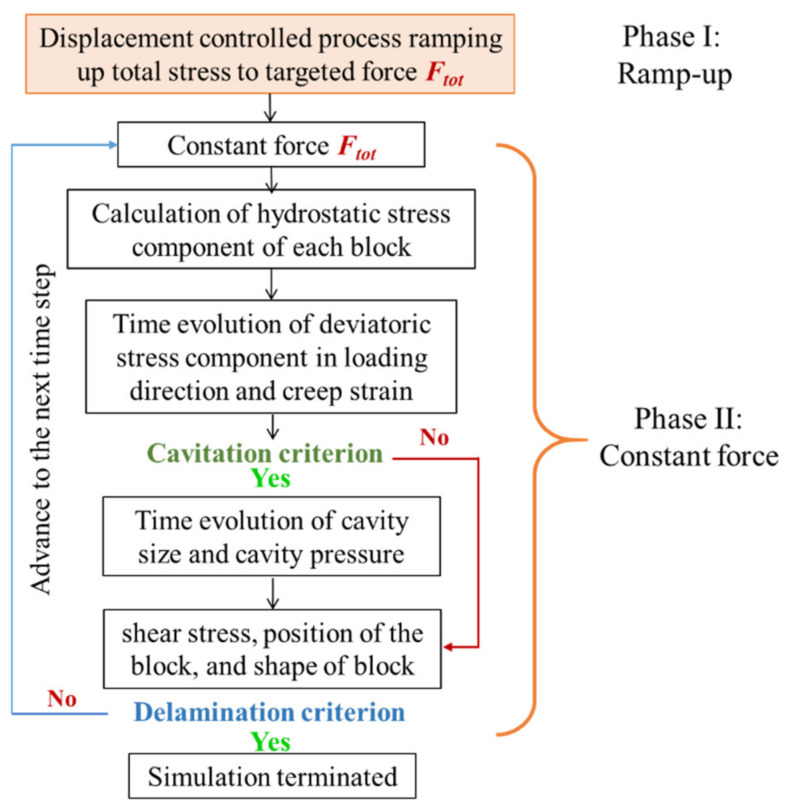
Modeling algorithm for creep of single-layered joint, showing flowchart for both phases of the creep test.

The PSA is lightly cross-linked and is modeled with a standard linear viscoelastic model, as discussed in our previous paper [16]. The stresses are obtained by combining the standard linear model and the Green Strain tensor. Unlike in the stress–strain model in our previous work [16], in the creep model, the model geometry evolution is driven by the applied macro-stress at the substrate boundary instead of the applied macro-displacement at the boundary. In other words, while the stress–strain behavior was modeled as a deformation-controlled process, the creep response is modeled as a force-controlled process. The creep tests conducted in this study consist of two phases: (i) a fast ramp-up of the stress to the desired level, followed by (ii) a steady dwell of the stress at a constant value. The detailed algorithm for creep model is shown as Figure 10.

**Figure 11:**
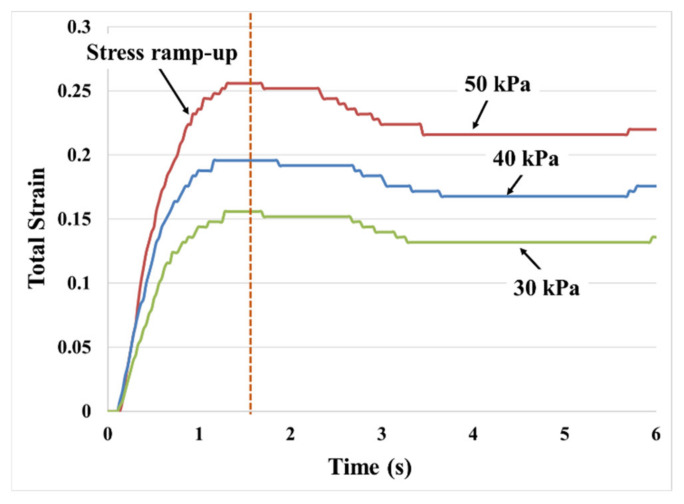
Creep stress ramps up to the pre-set steady-state value within the first 3 s of the creep test and is then held constant for the duration of the creep test (130 μm thick PSA).

**Figure 12:**
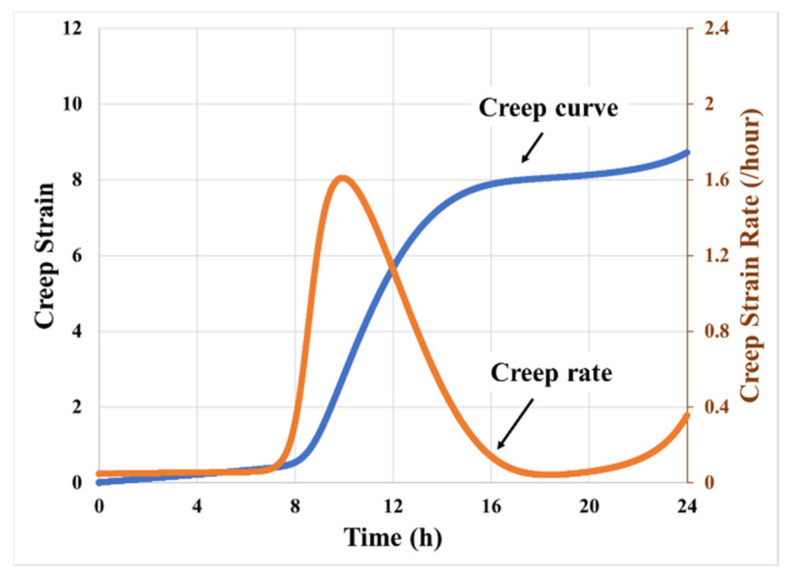
Model prediction of creep curve and creep rate curve of PSA bonded assembly at the stress level of 50 kPa based on the data collected in Table 1.

The resulting evolution of creep deformation is highly dependent on the history of the stress components. Therefore, to obtain the full history of the deformation process, the initial stress ramp-up process, shown in Figure 11, must be included in the simulation of the creep test. The stress ramp-up phase is modeled as a rapid displacement-controlled process and is finished within a very short time, using the stress–strain model [16]. Then, during Phase II (constant-stress creep phase), the total stress accumulated at the last step of Phase I is held constant. The algorithm for modeling the response to the creep is schematically shown in Figure 12. The ramp-up to the creep stress magnitude is modeled by a deformation-controlled stress–strain response. The stress ramp-up process is automatically terminated by the creep algorithm as the stress reaches the preset level for each creep test. This process governs the methods that are used to apply the external load or boundary condition.

The same standard linear constitutive model is used for the bulk PSA, both during the initial ramp-up phase and also during the subsequent constant stress creep phase. The constitutive equations for each phase (displacement-controlled ramp and stress-controlled creep processes) are shown in Equations (3) and (4), respectively:

(3) $${\dot{\sigma }}_{zz}^{d}=\frac{1}{\eta }\left(\frac{1}{2}G_{1}G_{2}\left(\lambda^{2}-1\right)+\left(G_{1}+G_{2}\right)\eta \left(\frac{2\dot{\lambda }}{\lambda }\right)+\eta \sigma_{zz}^{d}\left(\frac{2\dot{\lambda }}{\lambda }\right)-G_{1}\sigma_{zz}^{d}\right)$$

(4) $$\dot{\lambda }=\frac{\lambda }{2\eta \left(G_{1}+G_{2}+\sigma_{zz}^{d}\right)}\left[G_{1}\sigma_{zz}+\eta {\dot{\sigma }}_{zz}-\frac{1}{2}G_{1}G_{2}\left(\lambda^{2}-1\right)\right]$$

where $\sigma_{zz}^{d}$ is the *zz* component of the deviatoric stress tensor, $G_{1}$ is the modulus of the individual spring discussed in our previous paper on stress–strain response [16], $G_{2}$ is the modulus of the spring in the Maxwell element, and η is the viscosity of the dashpot in the Maxwell element. λ is the stretch ratio and $\dot{\lambda }$ is the loading strain rate of the PSA block.

### 3.2. Cavitation Criterion

The stress levels used in this study for creep testing are typically lower than those encountered in the stress–strain testing reported in our previous paper on stress–strain response [16]. However, plenty of cavities are still observed during the long-term creep deformation process. Therefore, in the creep model, the critical stress criterion is no longer suitable for modeling cavity initiation. Instead, the cavitation initiation is dominated by diffusive motion of polymer molecules—the bulk adhesive deforms under very low stress, while the cavities’ size slowly increases with the volume conservation of the bulk adhesive. Modeling trials reveal that in Phase II, a strain-based cavitation criterion performs better. The threshold strain value for this strain-induced creep cavitation model is deduced from the experimental results.

### 3.3. Cavity Growth Criterion

Cavity-induced creep deformation is best modeled with two separate deformation regions centered around a typical cavity. One is the far-field slow deformation region, which is in the bulk adhesive, far from the cavity. The other is the near-field rapid deformation region, which is in the region near the cavity, especially when the cavity is in the initial rapid growth stage. Therefore, we assign different viscosities, $\eta^{0}$ and $\eta^{\infty }$, for these two fields where superscript 0 represents relaxation time for the slow deformation region and superscript ∞ represents relaxation time in the rapid deformation region. Therefore, when cavities evolve by rapid expansion, ${\dot{R}}_{i}/R_{i}\gg 1/s$, the expansion rate of the *i*th cavity, $\dot{R_{i}}$, in a standard linear viscoelastic medium is defined by [21]:

(5) $$\dot{R_{i}}=\frac{R_{i}}{4\eta^{\infty }}\left(-P_{i}-\frac{2\gamma }{R_{i}}-\frac{G}{2}\left\{5-{\left(\frac{R_{0}i}{R_{i}}\right)}^{4}-4\frac{R_{0i}}{R_{i}}\right\}\right)$$

where γ is the surface tension of the adhesive, $R_{0i}$ and $R_{i}$ are the initial and the current cavity radius, respectively, and G is the equivalent shear modulus of the bulk PSA, defined as $G=\sigma_{zz}^{d}/3\lambda$.

When the cavity size is large enough, the growth rate of the cavity decreases. The deformation rate of the bulk adhesive in the near-field of the cavities becomes similar to that of the bulk adhesive in the far-field. This means ${\dot{R}}_{i}/R_{i}≅\dot{\lambda }/\lambda$, and the expansion of cavities is defined by [21]

(6) $$\dot{R}=\frac{R}{4\eta^{0}}\left(-P_{i}-\frac{2\gamma }{R_{i}}-\frac{G}{2}\left\{5-{\left(\frac{R_{0}i}{R_{i}}\right)}^{4}-4\frac{R_{0i}}{R_{i}}\right\}\right)$$

The growth of the interfacial cavity, which was extensively discussed in our previous publication [16], is controlled by the coefficient of friction between the fibril foot and by the surface free energy of the substrate. The non-monotonic effect of the surface free energy on the creep behavior is simply described by:

(7) $$\mu_{2}=a-\left|\gamma_{sub}-\gamma_{PSA}-0.02\right|\times 10^{7}$$

where a is the parameter to calibrate the coefficient of friction in the creep model, $\gamma_{sub}$ is the surface free energy of substrate, and $\gamma_{PSA}$ is the surface free energy of PSA.

### 3.4. Stress Component in Creep Model

The total stress during the stress ramping up process is:

(8) $$\sigma_{tot,z}=b{\bar{\sigma }}_{zz}+\left(P_{o}-{\bar{P}}_{cav}\right)\left(1-b\right)$$

where over bar stands for the average over all blocks, b represents the ratio of actual bonding area to the area of a substrate, which is discussed in Section 3 of our previous paper on stress–strain response of PSAs. If b>1, 1−b=0.

In the constant stress creep phase, the total stress is held constant from the last step of the stress ramp up of Phase I. Stress component ${\bar{\sigma }}_{zz}$ and its deviatoric part ${\bar{\sigma }}_{zz}^{d}$ at the bonding interface evolve as follows:

(9) $${\bar{\sigma }}_{zz}=\frac{1}{b}\left(\sigma_{tot,z}-\left(P_{o}-{\bar{P}}_{cav}\right)\left(1-b\right)\right)$$

(10) $${\bar{\sigma }}_{zz}^{d}=\frac{1}{2}\left({\bar{\sigma }}_{zz}-{\bar{\sigma }}_{xx}\right)$$

where, although the total force remains constant during phase II (constant stress creep process), the partitioning between the hydrostatic stress and the deviatoric stress continuously evolves, leading to the observed changes in the creep rate.

## 4. Simulation Results

Figure 12 shows the creep curve predicted by this model, for the parameter set from Table 2, for a constant loading stress of 50 kPa. It can be seen that during the initial stages of deformation, there is a steady creep rate. As the cavitation initiates (approximately at an engineering strain of 180%), we see a transient phase where the creep strain rate increases dramatically and reaches its peak within a very short period. This is attributed to the high hydrostatic release rate at the initial expansion stage of cavities—stress transfer from hydrostatic form to deviatoric form. Then, the hydrostatic stress release rate decreases as the size of the cavities increases and the amount of hydrostatic stress preserved in the bulk of PSA decreases. The creep strain rate diminishes correspondingly and stabilizes at a lower value close to that in the initial stage. The duration for this transient phase is about 20%, which is defined by the ratio between transition duration, 4 h, and duration of the entire secondary creep process, 20 h, while the corresponding creep strain accumulation during this transient phase is more than 70% of the total creep deformation. The total creep deformation is defined as the duration from the primary creep to the initiation of the tertiary creep, which is about 20 h. These trends of multiphase creep response are consistent with the empirical results reported earlier in Section 2.

In this model, due to the use of rate-dependent viscosity, the near-field viscosity in the vicinity of the cavities is much lower than the viscosity in the rest of the bulk far-field area. This value is highly dependent on the expansion rate at each time step. For simplicity, to fit experimental data, the near-field viscosity is 0.1% of the far-field viscosity for the duration of creep.

**Figure 13:**
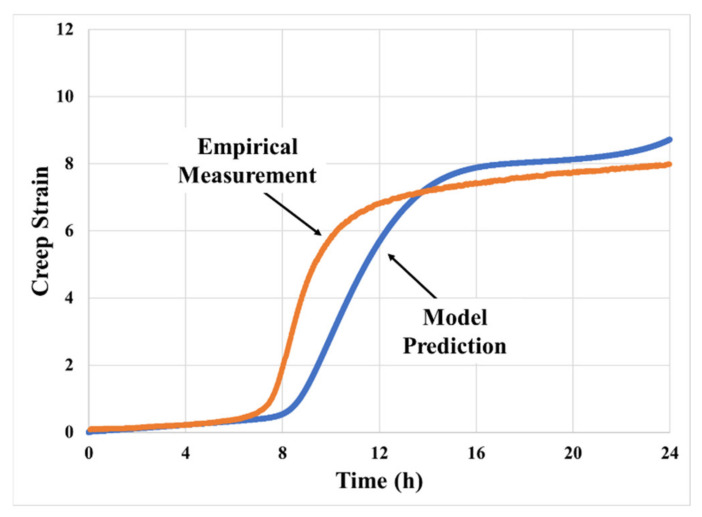
Empirical result vs. model prediction with identical parameters at a stress level of 50 kPa at 70 °C.

Figure 13 shows the quantitative comparison of empirical measurement and model prediction of the creep behavior with identical operating parameters. The model prediction first characterizes the multiphase secondary creep behavior. Moreover, it shows good agreement to some critical value in the empirical measurement, such as strain to primary transition, duration of the transition, and change of the strain rate. However, the transition from secondary creep to tertiary creep does not match very well.

### 4.1. Deformation and History of Stress Component

**Figure 14:**
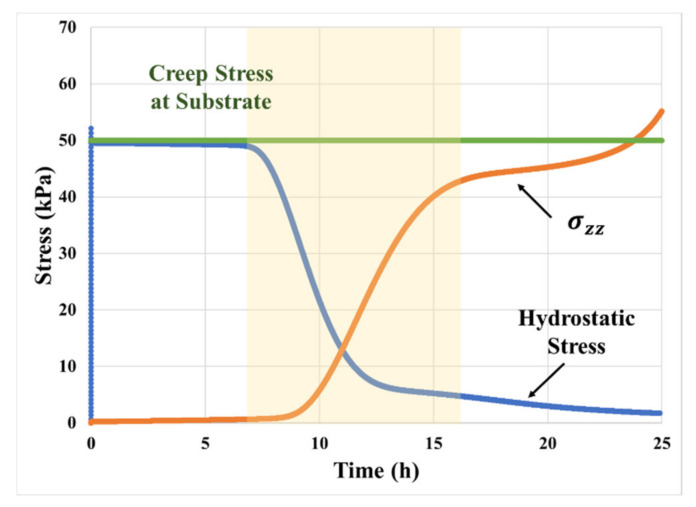
History of deviatoric stress (*z* component of the deviatoric part of the viscoelastic stress tensor) and hydrostatic stress in the foot area of PSA fibril.

The corresponding history of relevant stress components is shown in Figure 14. The loading stress is the average far-field stress applied on the bonding substrate in the *zz* direction and is maintained constant during the entire creep deformation. The hydrostatic part of the stress field dominates over the deviatoric part during the initial stages of the creep deformation due to the high geometric confinement. However, it starts decreasing as the stresses start relaxing due to cavity initiation. By the end of the transition phase, more than 90% of the hydrostatic stress is released. In contrast, the *z*-component of the deviatoric stress starts to increase as the cavitation initiates and the rate of increase slows down towards the end of the transition phase (shown by the shaded region in Figure 14). There is a noticeable increase in the *z*-component of deviatoric stress in the final stage of the creep response. This increment is due to the initiation of partial delamination between PSA fibril and bonding substrate. The delamination process decreases the actual length of the PSA fibril foot, thus increasing the actual stress acting in the adhesive fibril.

### 4.2. Effects of Loading Stress Level 

**Figure 15:**
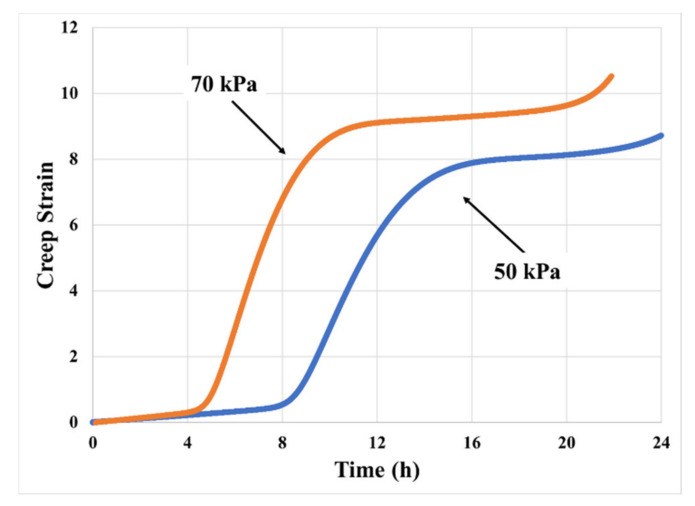
Effects of creep stress level on model predictions at 70 °C.

Figure 15 shows the effects of loading stress level on creep model prediction at 70 °C temperature. As expected, creep strain rates and accumulated creep strain are higher at the higher stress which is consistent with the experimental results shown in Figure 6. Higher stress level also causes earlier initiation of the transition during the steady-state creep. However, unlike the experimental data, the post transition creep rate does not increase significantly as the loading stress increased. This may be due to the assumption of constant interfacial friction coefficient between PSA and substrate used in the current model.

### 4.3. Effect of Adhesive Modulus and Viscosity 

**Figure 16:**
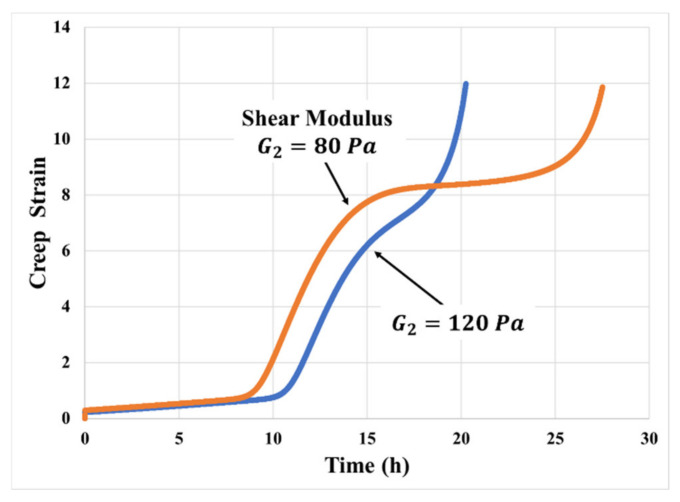
Effects of adhesive modulus on creep model prediction (50 kPa, 70 °C).

Different PSA materials have different moduli and viscosities. The mechanistic model is sensitive to both sets of adhesive material properties. Figure 16 clearly indicates that the creep curves for different moduli of the adhesive exhibit different degrees of transition and different total creep strain histories for a given stress and given temperature. Increase in the shear modulus $G_{2}$ of the adhesive leads to delay in the transition and a decrease in the deformation both during and after the transition in the creep response. The lower the modulus, the softer the material, and the more significant the strain expected within the transition. However, it takes a longer time to harden the material sufficiently to reach delamination stress at the adhesive-substrate interface.

**Figure 17:**
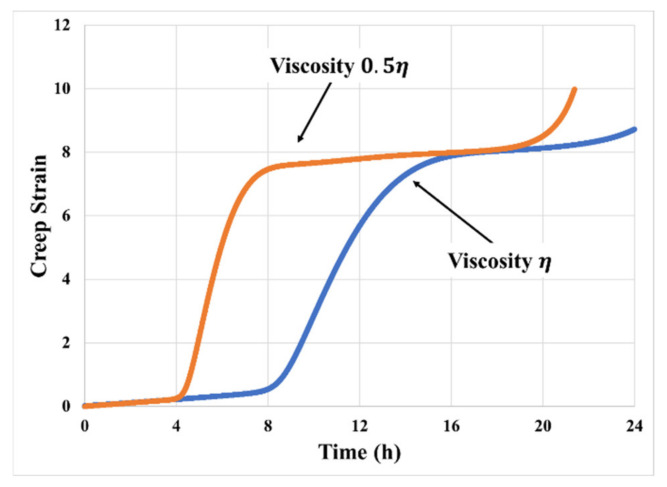
Effects of PSA viscosity (and stress relaxation time) on creep model prediction (50 kPa, 70 °C).

Figure 17 shows how the creep response depends on the viscosity. Increasing η delays the primary transition and decreases the peak creep rate during the primary transition. The decrease in the time span is due to the decrease in cavity growth rates with an increase in viscosity.

### 4.4. Effect of Substrate Surface Roughness

**Figure 18:**
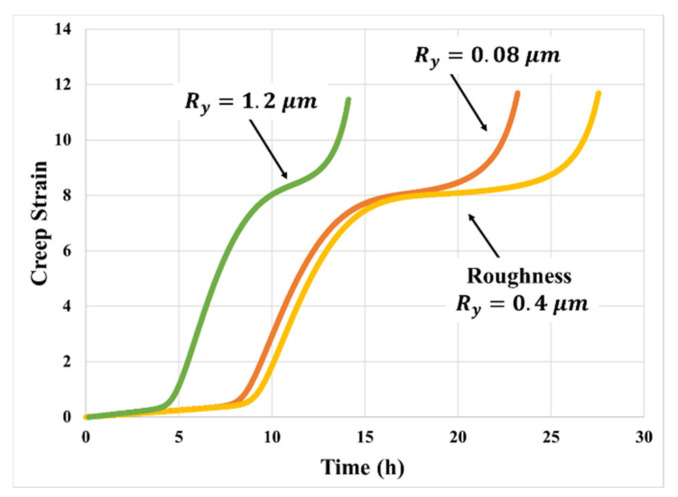
Effect of surface roughness on creep model prediction (50 kPa, 70 °C).

In our previous publication on stress–strain response of PSAs [16], the stress–strain response was shown to have a non-monotonic dependence on the bonding substrate surface roughness. The mechanistic enhanced “block” model includes the underlying physics using a simplified approach for capturing the effects of surface topology on (i) effective bonding area and (ii) initial interfacial defect size. The higher the surface roughness, the larger the effective bonding area and the lower the normal stress component at the interface, with direct consequences in suppressing interfacial cavitation and increasing creep resistance. Conversely, increasing surface roughness also raises the average characteristic size of initial interfacial micro-voids, thus promoting cavitation and diminishing creep resistance. The trade-off between these two competing effects results in non-monotonic dependence of creep deformation on surface roughness of the substrate, as shown in Figure 18. The part of model prediction of creep resistance can deteriorate as the substrate surface becoming too rough is evidenced by the empirical observation shown in Figure 7. However, empirical verification of this non-monotonic behavior is deferred to a future study.

### 4.5. Effect of Substrate Surface Free Energy

**Figure 19:**
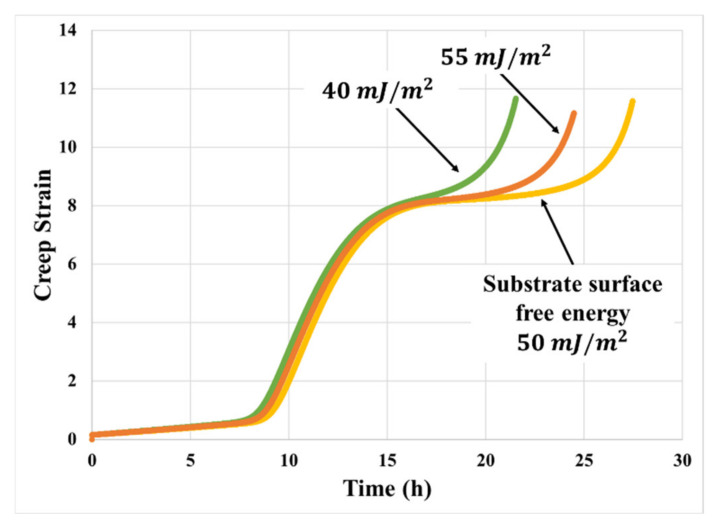
Effect of substrate surface free energy on creep model prediction (50 kPa, 70 °C with PSA surface free energy of 30 $\mathrm{mj}/m^{2}$).

The current study does not include experimental study of the influence of substrate surface energy on creep resistance. However, based on the empirical observations on the stress–strain performance and information from other studies [22], such dependency has been included in the predictive model. The simulation results, as shown in Figure 19, indicate that if substrate surface free energy is slightly higher than the PSA surface free energy, the PSA joint can achieve its maximum creep resistance. The adhesive wets better on the surface of substrates with surface free energy that is slightly higher than the PSA it bonds to; therefore, increasing the adhesion strength and interfacial mechanical locking and decreasing the chance of premature fibril foot delamination during the deformation process. Empirical verification of this non-monotonic behavior is deferred to future work.

### 4.6. Effect of the Adhesive Thickness

**Figure 20:**
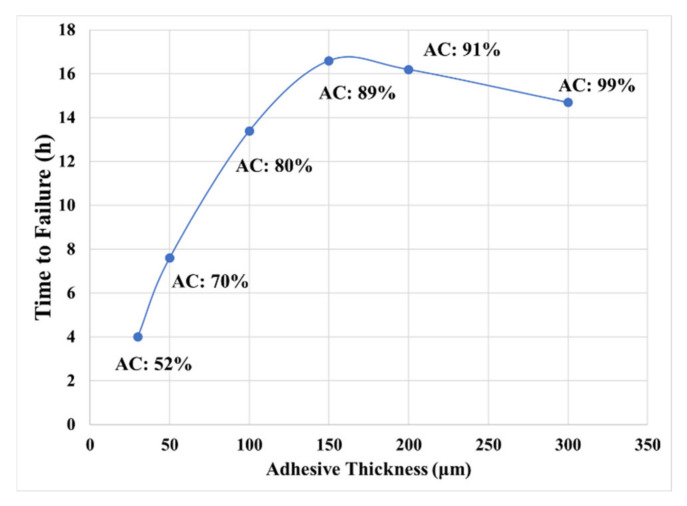
Effect of thickness on creep resistance (AC is the percentage of actual contact area defined by the bonding area divided by the substrate nominal area).

Figure 20 shows the capability of this model to predict the effect of adhesive thickness on the creep resistance. In this section, time to “failure” is defined as the time taken for the adhesive layer to deform to 1000% strain, under tensile creep load. As seen from the plot, time to “failure” has a non-monotonic dependence on thickness, increasing steeply when the adhesive is less than 150 μm thick (as demonstrated in Tordjeman’s [19] empirical results). However, when the adhesive layer becomes too thick (more than 200 μm), time to “failure” starts to decrease as the thickness of the adhesive increases. This non-monotonic behavior is the result of constant competition between an increase in effective bonding area (as discussed in our previous paper [16]) and a concurrent decrease of geometric confinement in the adhesive layer.

## 5. Limitations of the Current Study

The predictive mechanistic creep model discussed in this paper is capable of reproducing many features found in the experimental creep response of PSA-bonded assemblies, especially the multiphase nature of the creep response due to the constant competition between softening (due to cavitation) and hardening (due to fibrillation processes). However, some simplifications and approximations in the modeling algorithm (listed below) induce some discrepancies between the experiments and the model predictions.

(1) The actual cavitation mechanism is based on the balancing of stress level and the evolution of defect size due to the mass diffusion process. The model in this paper simplifies the physics of the phenomenon and uses the local deviatoric strain level as a proxy for the cavitation criterion.
(2) The interfacial friction and critical energy release rate are assumed to be linearly dependent on the surface free energy. This assumption is based on literature data reporting a linear dependence between peel-strength and surface free energy of the polymeric substrate.
(3) Some of the model predictions, e.g., the effect of adhesive properties (modulus and viscosity), have not been verified by empirical studies, due to the limitations of available test results. Additional verification of these model predictions will be conducted in future studies.
(4) Due to the complex stress distribution in the force-controlled creep phase, average stress is used for determining the evolution of the strain. In reality, the *zz*-direction deformation is the same for each fibril, but the stresses in each fibril will vary depending on the fibril location.
(5) Additional experiments are needed to understand the non-monotonic dependence on the substrate surface roughness and to investigate the effects of temperature-dependent material properties and interfacial interaction between PSA and substrate.
(6) The differences in the effective viscoplastic material properties between the near-field and far-field adhesive materials around a cavity are not clearly distinguished.
(7) The transverse stresses due to Poisson’s ratio mismatch between substrate and PSA are not explicitly included in the “block” model. Some inaccuracies of the model prediction may be caused by this inadequacy.

The predictive model in this study is only applicable for tensile (peel) loading, not for shear.

## 6. Summary

Multiphase creep response is observed when joints between rigid substrates, bonded with highly ductile single-layered PSA, are subjected to tensile (peel) loading. The transitions in the creep curve are the result of competition between mechanisms of softening due to cavitation and stiffening due to fibrillation, during the deformation and debonding process. The interplay of these mechanisms is highly dependent on the adhesive properties and surface properties of the bonding substrate.

A predictive mechanistic model, which is based on mechanisms of interfacial and bulk cavitation, the growth of cavities, fibrillation, and interfacial mechanical locking, is presented to capture the tensile creep response of single-layered PSA/substrate joints. This predictive model shares a similar modeling technique with the stress–strain model presented elsewhere [16] but uses a different algorithm due to the differences in the loading history (displacement-controlled vs. stress-controlled). The proposed creep model is capable of reproducing the multi-phase response of uniaxial tensile creep of single-layered PSA bonded with rigid substrates. Moreover, it can provide insights about the effects of PSA properties (modulus and viscosity) and substrate surface properties (surface free energy and roughness) on the creep response of PSA/substrate joint. Parametric studies using the predictive model are carried out to demonstrate the sensitivity of the creep model to different material and model parameters as well as the loading stress level.

## Figures and Tables

**Table 1:** Strain value to critical locations of the creep curve.

Critical Strain Value	Mean	SD
Initiation to transition	0.8	0.13
Peak of transition	2.7	0.08
End of transition	8.1	1.2

**Table 2:** Simulation parameters.

Parameter (Unit)	Value
PSA thickness $H_{0}$ (m)	$1.3\times 10^{-4}$
PSA width $W_{0}$ (m)	$7\times 10^{-3}$
Stress level (kPa)	50
Relaxation time τ (s)	8000
Elastic modulus $G_{1},\;G_{2}$ (Pa)	20, 80
Strain hardening coefficient *h*	2.2
PSA surface energy $\gamma_{PSA}$ (mJ/m^2^)	30
Substrate surface energy $\gamma_{sub}$ (mJ/m^2^)	50
Initial cavity $R_{y}$ (m)	$0.4\times 10^{-6}$
Roughness wavelength $X_{s}$ (m)	$5\times 10^{-6}$
Atmosphere pressure $P_{o}$ (Pa)	$10^{5}$
PSA penetration parameter m	60
Number of blocks	20
Time step (s) for stress ramp-up	0.02
Time step (s) for creep	20
Critical strain to cavitation	1.4
Friction parameter	$4\times 10^{7}$

## Data Availability

Data is contained within the article.
